# Native structure-based modeling and simulation of biomolecular systems per mouse click

**DOI:** 10.1186/1471-2105-15-292

**Published:** 2014-08-29

**Authors:** Benjamin Lutz, Claude Sinner, Stefan Bozic, Ivan Kondov, Alexander Schug

**Affiliations:** Steinbuch Centre for Computing, Karlsruhe Institute of Technology (KIT), Hermann-von-Helmholtz-Platz 1, 76344 Eggenstein-Leopoldshafen, Germany; Department of Physics, Karlsruhe Institute of Technology (KIT), Hermann-von-Helmholtz-Platz 1, 76344 Eggenstein-Leopoldshafen, Germany

**Keywords:** Protein folding, RNA folding, Native structure-based model, Molecular dynamics, GridBeans

## Abstract

**Background:**

Molecular dynamics (MD) simulations provide valuable insight into biomolecular systems at the atomic level. Notwithstanding the ever-increasing power of high performance computers current MD simulations face several challenges: the fastest atomic movements require time steps of a few femtoseconds which are small compared to biomolecular relevant timescales of milliseconds or even seconds for large conformational motions. At the same time, scalability to a large number of cores is limited mostly due to long-range interactions. An appealing alternative to atomic-level simulations is coarse-graining the resolution of the system or reducing the complexity of the Hamiltonian to improve sampling while decreasing computational costs. Native structure-based models, also called Gō-type models, are based on energy landscape theory and the principle of minimal frustration. They have been tremendously successful in explaining fundamental questions of, e.g., protein folding, RNA folding or protein function. At the same time, they are computationally sufficiently inexpensive to run complex simulations on smaller computing systems or even commodity hardware. Still, their setup and evaluation is quite complex even though sophisticated software packages support their realization.

**Results:**

Here, we establish an efficient infrastructure for native structure-based models to support the community and enable high-throughput simulations on remote computing resources via GridBeans and UNICORE middleware. This infrastructure organizes the setup of such simulations resulting in increased comparability of simulation results. At the same time, complete workflows for advanced simulation protocols can be established and managed on remote resources by a graphical interface which increases reusability of protocols and additionally lowers the entry barrier into such simulations for, e.g., experimental scientists who want to compare their results against simulations. We demonstrate the power of this approach by illustrating it for protein folding simulations for a range of proteins.

**Conclusions:**

We present software enhancing the entire workflow for native structure-based simulations including exception-handling and evaluations. Extending the capability and improving the accessibility of existing simulation packages the software goes beyond the state of the art in the domain of biomolecular simulations. Thus we expect that it will stimulate more individuals from the community to employ more confidently modeling in their research.

**Electronic supplementary material:**

The online version of this article (doi:10.1186/1471-2105-15-292) contains supplementary material, which is available to authorized users.

## Background

Great progress in experimental techniques, such as X-ray diffraction analysis and nuclear magnetic resonance spectroscopy, has led to a vastly increased diversity and quality of biomolecular structure data presented in the Protein Data Bank [1]. By combining this information with biomolecular simulation one can supplement static structural models with an increasingly detailed dynamic picture even for huge molecular machines like the ribosome [2, 3]. Still, exploring the dynamical nature of molecular life poses a significant challenge for present-day computational resources. While astonishing progress in this field has led to first all-atom protein folding simulations for small globular proteins on the millisecond timescale [4], the required specialized supercomputers are not publicly available.

An intriguing alternative to simulating these biomolecules with atomic resolution in physics-/chemistry-based forcefields is focusing on their essential features and coarse-graining (CG) either the resolution of the system or its forcefield [5, 6]. In such CG models, the granularity of the system is typically changed from an all-atom representation by mapping groups of atoms into single beads. This reduces the computational complexity and provides access to longer timescales and length scales. For example, in the MARTINI forcefield typically four heavy atoms and the associated hydrogens are mapped into a single bead representing the respective group [7]. Another approach, the so-called native structure-based modeling (SBM) [8–11], is based on energy landscape theory and the principle of minimal frustration [12]. Accordingly, the energy landscape has a funnel-like shape biased towards the native state of a protein. In a long evolutionary process, the energy landscape was smoothened by minimizing (energetic) roughness and frustration to enable efficient folding by ensuring a dominance of native interactions over non-native ones. Thus the structural information of the native state becomes an integral part of the model potential describing the interactions in the biomolecular system. Topological information, such as the contact map of the native state, is usually employed (Gō potentials [13]) initially within coarse-grained C _*α*_ [14] or C _*β*_ [15] models and more recently, within all-atom models [16]. The introduced bias towards the native state reduces the forcefield complexity without loss of essential information and enables the simulation on biologically relevant timescales, e.g. protein folding simulation [16] on the all-atom level on standard desktop computers. In many recent studies of protein dynamics SBM has become the tool of choice to rationalize experimental observations by means of computer simulations [17]. Structure based modeling provides now an established method for physical understanding of, e.g. folding pathways [14, 18], folding kinetics [19], effects like posttranslational modifications [20] or oligomerization [21]. The SBM has been generalized to describe also proteins with two or more stable native conformations [22] in order to model functional transitions, e.g. allostery and ligand binding. Moreover, transition states, which are experimentally directly not accessible, have been studied using SBM [23, 24]. Still, one might easily expect that focusing on native interactions and neglecting non-native interactions within SBM distorts simulation results. Significant effort has therefore been invested to examine to which extend non-native interactions play critical roles [17, 25–28]. In particular, recent work has carefully analyzed the role of native interactions in prior atomically resolved simulations [29, 30]. These studies have found dominance of native interactions to non-native ones and good agreement between results from CG SBM simulations and more fine-grained models [31]. Overall, SBM is accurate for the extreme case of minimal or no frustration, with all-atom models realizing all possible non-native interactions [32]. Somewhat surprisingly, neglecting non-native interactions does not significantly distort simulations results [17, 31]. This not only makes the minimalistic SBM very useful, but also suggests that naturally occurring proteins seem to possess low frustration [8, 33].

In numerous close collaborations between experimental and modeling research groups the SBM methods are applied [34–36]. To enable regular use by the community of, in particular, experimental scientists or other researchers who do not possess specialized programming and/or modeling experience, it seems senseful to establish a research infrastructure (similar to the PDB service) standardizing and simplifying the simulation setup and submission, as well as the evaluation of these simulations. This infrastructure should include services for development of novel models and adaption of existing models to new applications, and routine deployment of ready-to-go models. A first effort to establish such a service is the SMOG (Structure-based MOdels in GROMACS) web server [37] that is publicly available under http://smog-server.org/. This server provides a convenient setup of native structure-based simulations with several options for custom forcefield choice and parameterization. Going beyond mere folding, eSMBTools [38] is a Python-based toolkit allowing the simplified setup and evaluation of native structure-based simulations for proteins and RNA. The focus of this toolkit is enhancing these simulations by experimental or bioinformatics derived data, e.g. to enable the prediction of protein complexes or active conformations based on the statistical analysis of existing sequence databases [34, 39] or riboswitch folding [40]. In particular, forcefields and topologies of amino and nucleic acids are encoded in XML files making the toolkit easily extensible to other biomolecules, such as ligands.

Providing a modeling and simulation service for SBM solves several challenging issues which we outline in the following: i) The simulations require use of computing resources which are usually unavailable locally and the scientist has to face the high technical complexity of distributed computing infrastructure. To this end, several solutions providing access to remote computing resources already exist [41–43]. However, while hiding the complexity via virtualization of resources and abstraction, these middleware services are rather generic so that their direct use without the integration of the biomolecular model can pose even higher barriers for the end-user. ii) Multiple program codes (steps) have to be linked together in one composite application (workflow) via standard interfaces for automatic execution. Data sources and sinks at different workflow steps have to be linked via standard interfaces (dataflow). The existing solutions do not use generic standards (such as for example web services) but rather domain-specific solutions which have to be laboriously adapted to every new model and simulation. Currently, there are many program codes that do not blend in with each other and therefore efforts have been recently spent to partially alleviate this problem [44–46]. iii) The elements of the infrastructure exposed to researchers have to be reduced to minimum and made available via a modern graphical user interface (GUI). The challenging aspect is here the design decision what has to be included in the interface rather than the GUI implementation itself. The access to more functions improves the tool capabilities and flexibility but heightens the expert level.

In previous work, many of these issues have been tackled effectively for applications in high-throughput virtual screening [44], materials science [45, 47] and biomolecular NMR [46]. In particular, a data model based framework for data exchange between workflow steps has been proposed and a toolkit has been provided for automatic generation of a data access service for scientific applications [48]. Thereby, the issues outlined above can be treated by means of modern technologies such as web services and model-driven engineering leading to complete automation of the program interfaces, workflows and dataflows. A common approach which has been extensively applied is the science gateway (also known as web portal). For example, the virtual research community WeNMR has a large collection of portals providing production services for different applications in structural biology [49], including molecular dynamics simulations with Gromacs [50]. Data exchange in multi-step molecular simulations and analysis has been treated in several works previously [51–54]. Within the MoSGrid project the molecular simulation markup language (MSML) [51, 52] has been developed employing the concept of Chemical Markup Language (CML) dictionaries and used in quantum chemistry, molecular dynamics and docking simulations on the MoSGrid portal [55]. The Collaborative Computing Project for NMR (CCPN) [53] has provided a software framework that consists of a data model [54], the CcpNmr Analysis program, and a collection of additional tools, including the CcpNmr FormatConverter. The CCPN application programming interface (API) is available in three programming languages (C, Python and Java) and enables the integration of additional analysis and simulation software to build complex workflow applications.

In this paper, we present a software infrastructure which deploys SBM on distributed high-throughput computing (HTC) and high performance computing (HPC) resources providing a powerful interface for model development and user-friendly interface. The software provides a simple and still flexible graphical user interface for eSBMTools to allow end users to run SBM simulations without developing IT technical skills.

## Implementation

We have adopted the principles of Service Oriented Architecture (SOA) [56] to design the implementation of the platform. Thus, many of the generic components required for the implementation are available in existing and well established grid and cloud middleware stacks, from which we have selected the UNICORE middleware [41]. UNICORE is a fully fledged and mature open-source middleware which has been deployed and supported on large computing infrastructures such as PRACE (http://www.prace-ri.eu/) in Europe and more recently also on XSEDE (https://www.xsede.org/) in the USA. Computing clusters and other HPC resources managed by common batch systems, such as SGE, LSF, PBS Torque and LoadLeveler, can readily be used with UNICORE. Currently, UNICORE offers four different client variants: command line client, graphical rich client, a portal client and a high-level API. The UNICORE Rich Client (URC) [57] provides us with the software basis to integrate eSBMTools within an application extension called GridBean [58]. In addition, UNICORE includes a workflow engine and a powerful graphical workflow editor, completely integrated in the service layer and the URC, respectively.

Furthermore, there are several alternative open source middleware solutions. In the following, we briefly review two of them. The Globus Toolkit [42] is a widely used open source toolkit which implements numerous standards (for example web services) and allows building infrastructures for grid (internet) computing [59]. It has overall standards conformity similar to that of UNICORE but does not provide graphical clients and portals and can be used with the Galaxy workflow system [60]. The middleware ARC (Advanced Resource Connector) [43] has been developed and included in the software stack of the European Middleware Initiative. It implements web services standards for server-client communication and provides a GUI client. ARC integrates with the third-party workflow engine Taverna [61].

### Implementation architecture

**Figure 1:**
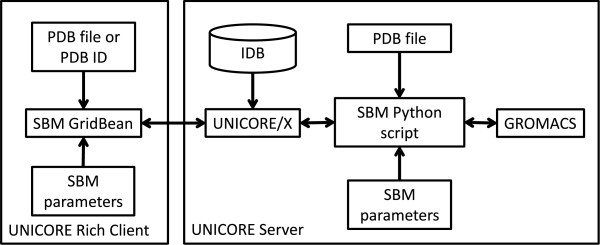
**General architecture of SBM simulation with eSBMTools based on UNICORE.**

Figure 1 shows the overall architecture of our eSBMTools integration. The main component is the SBM GridBean, a Java-based component, that captures and validates the SBM simulation parameters provided by the user via a GUI. The URC is the runtime container for the GridBean and provides the basic functionality to access UNICORE services e.g. for submitting and monitoring jobs, managing file storage and handling authentication and authorization.

The implementation of the SBM GridBean is based on the GridBean API [58] and consists of three major parts:

- A configuration file gridbean.xml which defines runtime parameters for the GridBean in the URC, e.g. names and versions of the GridBean and of the target application on the server;
- A Java class containing the GridBean model which defines the job parameters and input/output files;
- A plugin class which defines the graphical user interface and the mapping of the input components to the GridBean model. Here, a validator can be defined which performs type and plausibility checks on input parameters.

During job submission the URC translates the input from the SBM GridBean (model parameters, files, variables, required computing resources) into a JSDL (Job Submission Description Language) request which is then sent together with a PDB input file or a PDB ID to the UNICORE server. The UNICORE server has an incarnation database (IDB) which determines how to handle the incoming JSDL request. The IDB includes entries for all applications that are available to the URC for job submission. The IDB entry for the SBM application defines the Python interpreter as job executable and several parameters to configure the SBM Python script which is introduced in the next subsection.

### SBM Python script

The SBM Python script is based on the Python toolkit eSBMTools [38] that provides a wide range of functionalities to setup and manipulate structure-based models and to evaluate simulation output. Along with modular functions, Python is platform-independent and readily available on most HPC systems. Therefore it is an excellent choice for the functionality that eSBMTools is aiming for. The script consists of various preprocessing and post-processing modules that include functions called by a central Python script. This Python script represents the functional core unit of the SBM GridBean. The toolkit interfaces with GROMACS, a molecular dynamics software package [62] in a version provided by the SMOG homepage [63] that features an extension called g_kuh. The GROMACS extension g_kuh calculates the number of formed native contacts within the structure for each dumped frame of the simulated trajectory. The number of formed native contacts is often referred to as the Q value.

**Figure 2:**
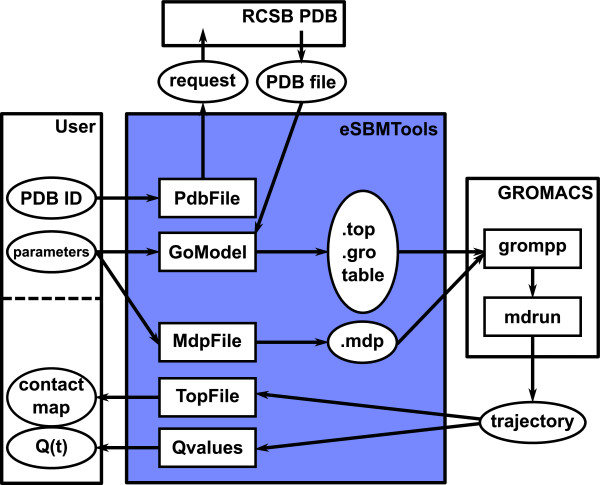
**Functionalities of the SBM Python script.**

Figure 2 shows a block diagram that depicts the utilized functionalities of the SBM Python script. The user provides a PDB ID [1] of the protein of interest and simulation options. The PDB ID is passed to the module PdbFile that prompts an according coordinate file download from the database. The PDB coordinate file and the provided options are processed by module GoModel that generates the required files for an SBM forcefield and atom coordinates in supported GROMACS file formats. Simulation options, e.g. temperature, simulation steps, random generation seeds, etc., are passed to module MdpFile that generates the simulation configuration file accordingly. After the GROMACS simulation the protein’s trajectory is evaluated by modules TopFile and Qvalues that create plots of the protein’s contact map and Q values trajectory, respectively. The contact map is a two-dimensional representation of the residue-residue contacts present in the native conformation and the Q value trajectory is the temporal evolution of the number of formed native contacts along the simulated trajectory.

## Results and discussion

### The SBM GridBean

**Figure 3:**
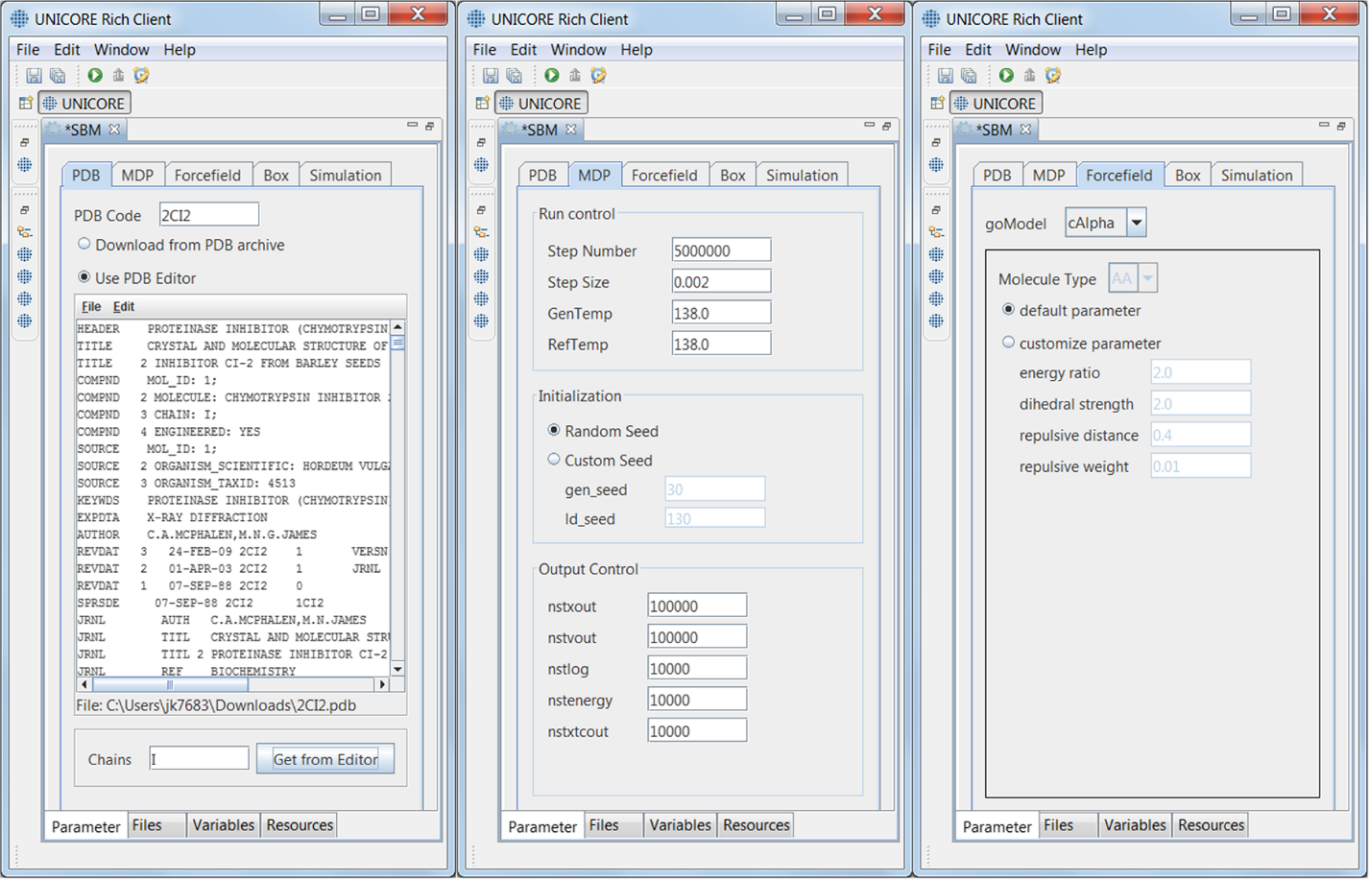
**Screenshots displaying the input parameter tabs “PDB”, “MDP” and “Forcefield” (from left to right, respectively) of the graphical user interface of the SBM GridBean.** Only the GridBean view is displayed, all other views of the URC (including the Grid Browser) are minimized.

Based on the SBM Python script (see previous section) we have developed an SBM GridBean that allows users to configure and run SBM simulations. Using UNICORE technology, we do not have to handle user authentication and authorization, web service protocols for job submission or file protocols during development but can rather concentrate on building an intuitive GridBean GUI which provides input fields for several methods and parameters for eSBMTools. The GridBean also validates all values entered by a user before sending them to the server. The GUI contains several tabs which group the parameters together (see Figure 3). In the following we outline these tabs.

#### PDB parameters

The PDB tab (see Figure 3, left screenshot) specifies the molecular structure to be analyzed. The user has the choice to specify a PDB ID in a text field. The PDB file with the actual molecular structure data is then downloaded by the SBM Python script. Alternatively, a PDB file which is available on the local file system can be specified. During job submission the PDB file is copied to the UNICORE server. In both cases the user can select the protein chains which should be processed by eSBMTools. By default all chains are selected.

#### Molecular dynamics parameters (MDP)

The MDP tab, shown in Figure 3 in the middle, offers more general parameters to control the mdrun program. The tab is structured into the three categories “Run control”, “Initialization” and “Output control”. These parameters are mapped by the Python script to an *.mdp file which forms the input for the GROMACS preprocessor grompp.

#### Forcefield parameters

The “Forcefield” tab defines specific parameter regarding the forcefield of an SBM simulation. The user can choose between a coarse grained *C*_*α*_ or an all-atom model which has influence on the precision and the runtime of the job. At the all-atom level the user has the ability to choose between amino acid (AA) and nucleic acid (NA) as molecule type. Depending on the chosen molecule type the corresponding topologies of molecular building blocks for proteins (AA) or DNA/RNA (NA) are used. A screenshot of this tab can be seen in Figure 3, on the right.

#### Box parameters

The distance between the outer most atoms of the molecule and the rectangular simulation box in all three dimensions can be adjusted on this panel.

#### Simulation parameters

The simulation tab allows to specify whether the structure-based model is only prepared and returned or a simulation with the structure-based model, i.e. the actual simulation is started on the computing resource (such as an HPC cluster) attached to the UNICORE server. In the first case the SBM Python script will create only the input files *.mdp, *.gro and *.top files and in case of a *C*_*α*_ simulation the file table.xvg. Creating only the input files is useful for computing sites where GROMACS is not available or where the system resources are limited to perform a computationally demanding mdrun. The created simulation files can then be transferred to a more capable computing site with a GROMACS installation. In the second case the SBM Python script creates all configuration files and calls grompp and mdrun. Both GROMACS commands are started as separate processes. The results of this simulation type are plots of the contact map and Q values as function of time.

### Further functions implemented and used in URC

#### Monitoring

An important feature of the URC is the Grid Browser with which the status of submitted simulations can be monitored. For every submitted simulation (single job or workflow) a working directory is created. This directory is the execution environment of the SBM Python script and contains the generated simulation files and the output of grompp and mdrun. The files in the working directory can be viewed and downloaded within the Grid Browser.

#### Update site

Another benefit of using UNICORE is the straightforward installation of GridBeans into the URC. As the URC is based on Eclipse it comes with an integrated update mechanism. For instance, the SBM GridBean is installed by specifying the URL^a^ of the project’s update site and following the instructions of the setup wizard.

#### Integration of third-party libraries

By using the Java based GridBean API and Eclipse as base technologies for the SBM GridBean we have integrated further Java libraries from the domain of bioinformatics into the SBM GridBean, for example the Jmol [64] library for visualization tasks. The structure of the simulated PDB or the trajectories from GROMACS are visualized with Jmol. BioJava [65] is another library that we have integrated into the SBM GridBean. Local PDB files (from the PDB tab) can be parsed and the value of the chains parameter is then automatically filled in using BioJava.

#### Workflows

GridBeans are reusable components which can be integrated into composite models in the form of UNICORE workflows [57, 66]. With the graphical workflow editor, which is standard component in the URC, a graph can be built specifying the execution order (control flow) of the simulation steps using several different GridBeans from the “Applications” pane of the URC. An output file of a GridBean can be transferred as input file to another GridBean (dataflow). The job submission and the file handling is done automatically by the UNICORE workflow service. In the next section we introduce a detailed case study of a workflow that includes the SBM GridBean.

### Exemplary workflow (case study)

**Figure 4:**
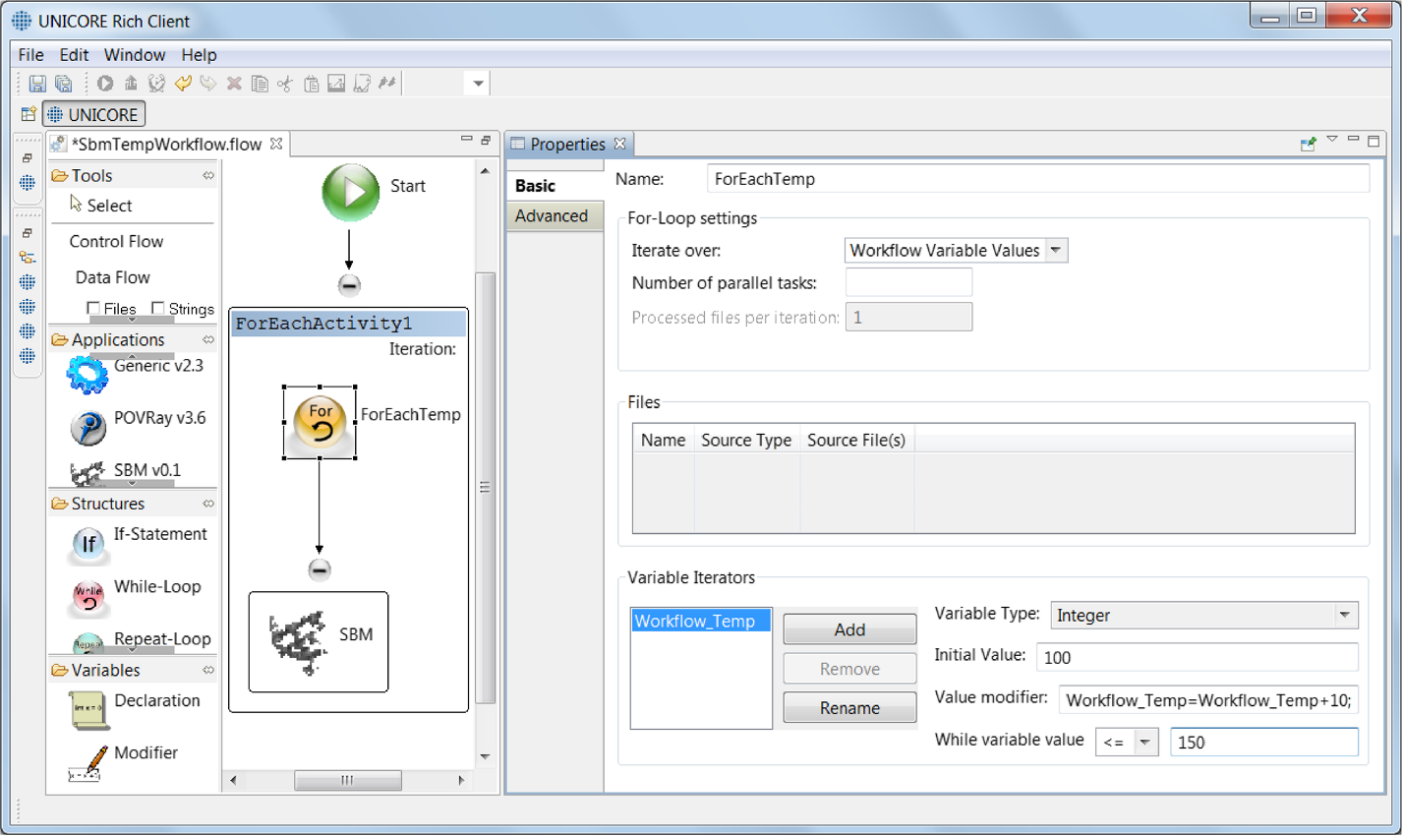
**Workflow for finding the protein’s folding temperature which is constructed in the workflow editor of URC employing the SBM GridBean.** The setup of the *foreach* control structure used for running several SBM simulations for different temperatures is done in the “Properties” tab, displayed on the right. Only the workflow editor view of the URC is shown.

**Figure 5:**
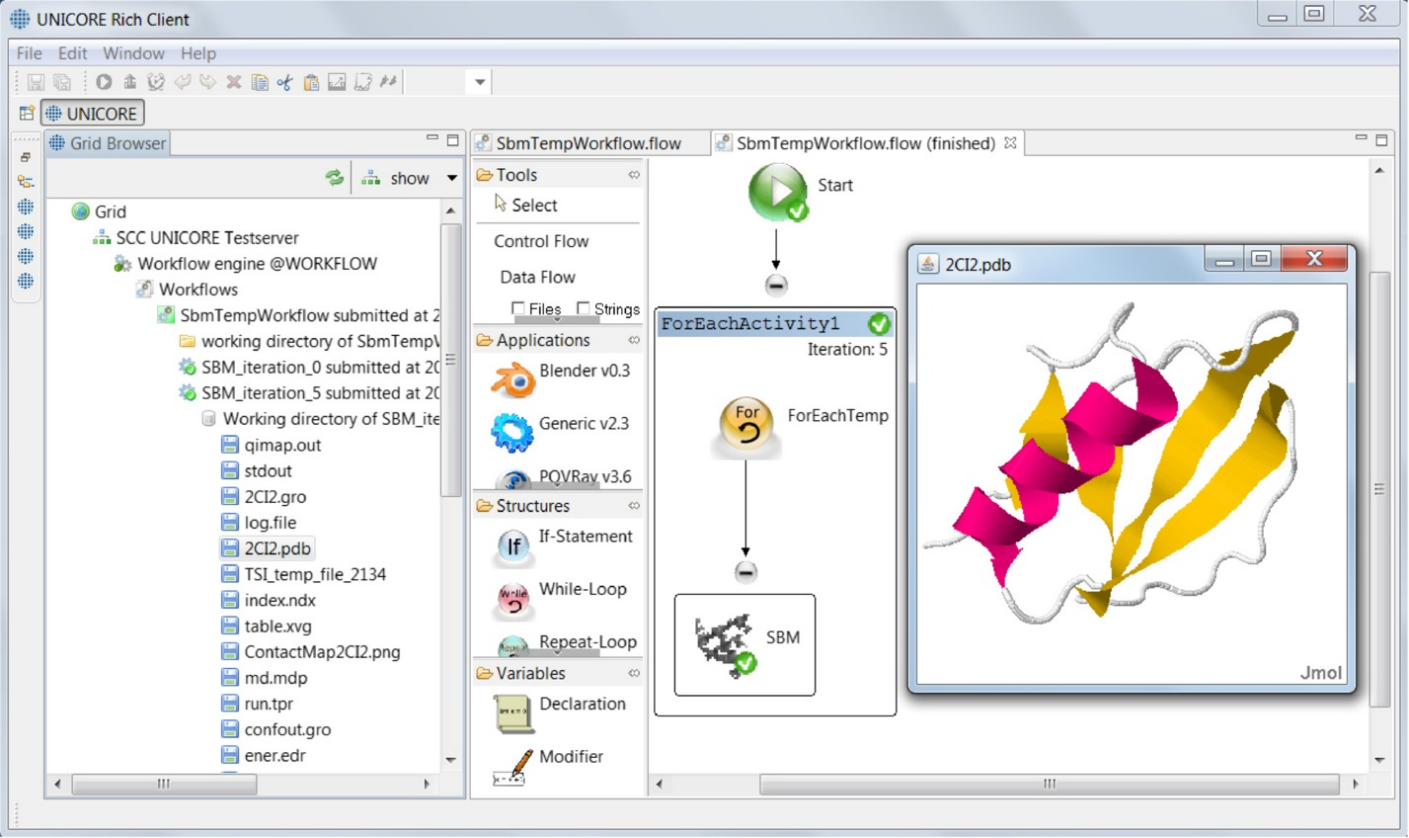
**The Grid Browser and further plugins, components of the URC, allow showing the progress and the results of the simulation.** On the left pane the GridBrowser is displayed, in the middle the finished workflow and the Jmol in the small window on the right.

**Figure 6:**
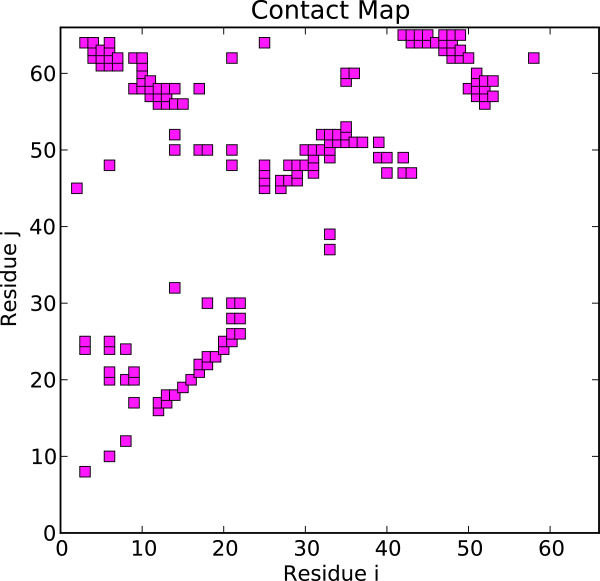
**Contact map of the serine proteinase inhibitor CI-2 with PDB ID 2CI2.**

**Figure 7:**
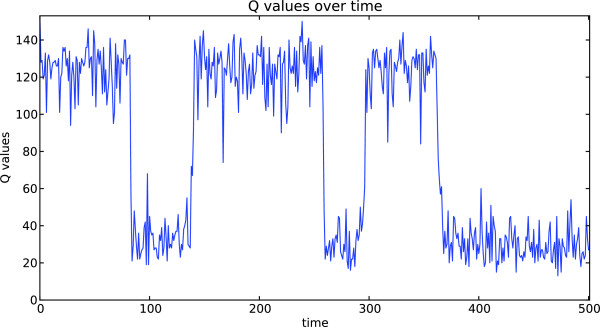
**Q value trajectory of a simulation for the protein CI-2.** The transitions between Q ≈ 40 and Q ≈ 120 indicate folding and unfolding events.

To provide a concrete application example that employs the developed SBM GridBean, we present a case study of protein folding dynamics of the before described application scenarios within a basic UNICORE workflow. We process ten exemplary proteins (PDB IDs 2CI2, 1G6P, 1ENH, 1SHF, 2QJL, 1RYK, 1RIS, 1BTH, 1TEN, 1MJC) containing from 45 up to 99 amino acids and both pure alpha-helical and mixed alpha-helical/beta-sheet structures, giving a cross-sectional overview. The SBM GridBean facilitates the setup and execution of an MD simulation in GROMACS and two exemplary evaluation steps at a specific temperature: A contact map is generated and the Q values along the simulated trajectory are calculated. To this end, the SBM GridBean is embedded in a *foreach* workflow control structure (see Figure 4) which automatically executes this step for each temperature. An analysis like this can be used, e.g., in algorithms that search for folding temperatures of proteins. In this study each protein was simulated at six different temperatures (100, 110, 120, 130, 140, and 150 in reduced GROMACS units, see the “Properties” tab in Figure 4) which enclose the region of expected folding temperatures in the present SBM parametrization. The folding temperature characterizes the temperature at which folded and unfolded conformations are equally occupied during a simulation. The constructed workflow is submitted and the simulation progress is monitored in the Grid Browser shown in Figure 5 on the left. The Jmol molecule viewer (see Figure 5 on the right) and further Eclipse plugins, that are integrated in the URC, allow visualization of the simulation results. For each temperature the workflow generates the contact map of the protein and a plot of the Q value trajectory as a function of time, depicted in Figures 6 and 7, respectively. The contact map gives detailed structural information about the protein’s native state. Based on the Q value trajectory it is possible to estimate whether the protein is in its folded or unfolded state at the simulated temperature.

The case study demonstrates the practicability of the presented SBM GridBean in operation on 10 exemplary proteins. The GridBean provides reusability for arbitrary protein structures at desired temperatures which allows its direct integration into workflows. The end user is not confronted with the details of the model or the implementation itself but can focus on the design and execution of the desired studies. The technical challenges are transferred to a developer who has carried out the required core implementation (SBM GridBean, SBM Python script). This core implementation needs to meet the requirements of projected workflows for which it might be beneficial in the future to split up the GridBean in parts dealing with pre- and post-processing.

In the Additional files we provide a screen dump showing the installation and setup processes (Additional file 1), as well as the usage of the SBM GridBean for the case study discussed above (Additional files 2 and 3). In this case study, we make use of the pilot service that is currently available for employees and students at KIT. In future, we plan to provide such a service for broader community as part of e-infrastructure projects.

### Benefits and drawbacks

In the following we will provide an outline of the major benefits of using our proposed software tools combined with a critical discussion of the drawbacks, particularly in comparison with existing alternative solutions.

The GUI of the SBM GridBean provides intuitive access to the most common methods of the eSBMTools modules and enables a wide range of individuals to run SBM simulations. While an end-user of the SBM GridBean are not faced with any line of code, the flexibility of changing the internal logic of the simulation steps is limited compared to the usage of the eSBMTools API directly. Thus, some variations of the model would require relevant changes in the SBM Python script. Nevertheless, this restricted flexibility has an additional advantage because the GUI does not expose well documented and validated features for changes the end-user. This increases the overall quality and reproducibility of the simulation output.

Generating the input files for GROMACS via a web server is a useful approach. The software must be installed only once on the web server and is then accessible from all over the world. By using the SBM GridBean the user has to additionally install the URC on their local desktop and the SBM GridBean into the URC from an update-site. If the web server is not attached to a computing cluster it may have limited resources for MD runs. In these cases, the prepared input files can be transferred from the web server to a more capable computing infrastructure that provides generic services for MD simulations with GROMACS. However, the UNICORE service comes with an integrated solution to access modern HPC and HTC computing infrastructures and is not only capable to prepare the input files for the simulation but also to efficiently execute computationally demanding all-atom SBM simulations using a massively parallel version of GROMACS. In all cases, the end-user will benefit from the uniform environment for modeling and simulation setup provided by the URC and the SBM GridBean.

Users who use a web server are supposed to trust the service providers in respect of handling their data. In addition to encrypting the whole client-server communication via SSL, the middleware UNICORE uses X.509 certificates for authentication and thus can ensure that only authorized persons have access to the connected resources. While contributing to the overall security substantially, managing X.509 certificates is considered generally more complex compared to simple user credentials such as username and password which are currently not supported. We expect that in future UNICORE will provide alternative authentication mechanisms.

In the following, we compare our proposed new software to an established tool in the community, particularly to the SMOG server, which was already introduced above. Except for the source code extensions to GROMACS it is a closed-source system leading to different concepts for establishing trust relations with their end-users compared to an open-source product. Furthermore, the extension of the platform with further functionalities, e.g. connecting to computing resources, and the setup an own instance of the service is not possible. In contrast, the eSBMTools API and the SBM GridBean are open source. Interested parties (end-users but mostly service providers) can download, adapt, redistribute and productively use the source code for their purposes.

The eSBMTools API and the SBM GridBean make use of several well known and tested bioinformatics libraries such as numpy, biopython, Jmol etc. These third-party libraries are well tested and have a high quality by permanent observation and development within the community. Using them increases the quality of the software and enriches it with many useful features for the end-user. Although Java and Python are used as the programming languages for the implementation of eSBMTools and the SBM GridBean, no programming language knowledge is required for using the SBM GridBean in the URC for constructing workflow models and running simulations.

The functionalities for constructing and executing workflows using UNICORE enables the design of individual custom-made projects employing SBM of biomolecular systems. The laborious working steps and protocols, as well as security mechanisms are hidden in the inner logic of the URC, the SBM GridBean and the UNICORE service and only properties and functions relevant for modeling and execution of workflows are exposed through the user interface so that end-users can focus on solving domain-specific challenges in biophysics, biochemistry or bioinformatics.

In Table 1 we summarize the benefits and potential drawbacks of our implementation of SBM compared to the SMOG server, eSBMTools and the SBM GridBean, as discussed above.

**Table 1:** **Comparison between SMOG server and SBM GridBean**

Feature	SMOG	eSBMTools	SBM GridBean
	server		
Graphical user interface	Yes	No	Yes
Access via web browser	Yes	No	No
Access via web server	Yes	No	Yes
Access to distributed	No	No	Yes
computing resources			
Use of X.509 certificate	No	No	Yes
Open source	No	Yes	Yes
Integration of third-party	?	Yes	Yes
libraries			
Provide means for	No	Yes	Yes
custom modeling			
Integration in composite	No	Yes	Yes
models			

## Conclusions

Significant progress on the technological side and the development of increasingly accurate forcefields enable biomolecular simulations which provide atomically detailed insight into the molecular machinery of life, yet require expert knowledge for the setup and analysis of data. One common class of such biomolecular simulations, native structure-based or Gō-type models, contributes to answer questions ranging from protein and RNA folding to function and structure prediction. We have developed a framework to facilitate construction and execution of workflows for these simulations based on the UNICORE middleware. We showed the straightforward setup of an exemplary workflow and expect that it can be adapted to individual projects as a service for the biomolecular simulation community.

## Availability and requirements

- **Project name:** UNICORE based integration of eSBMTools
- **Project home page:** The home page of eSBMTools is http://sourceforge.net/projects/esbmtools. The source code of the SBM GridBean is available under
- http://www.multiscale-modelling.eu/svn/esbmtools/gridbeans.
- **Operating system(s):** Platform independent
- **Programming language:** Java and Python
- **Other requirements:** UNICORE (version 6) server is required on the server host, URC (version 6) on the client host, Java Runtime Environment on both the client and server hosts, and Python interpreter and GROMACS on the computing resource.
- **License:** FreeBSD license (2-clause BSD license) for the SBM GridBean (Java source code) and GNU GPL (General Public License) for eSBMTools (Python source code)

## Endnote

^a^ For this project, the public update site is http://www.multiscale-modelling.eu/update-site/esbmtools/0.1/.

## Electronic supplementary material

Additional file 1: **Demonstration of installation and usage of the SBM GridBean: Part 1.** This video shows how to add an UNICORE site to the Grid Browser and install the SBM GridBean from an Update Site. (MP4 13 MB)

Additional file 2: **Demonstration of installation and usage of the SBM GridBean: Part 2.** This video shows how to construct a workflow for finding the folding temperature of a protein. (MP4 14 MB)

Additional file 3: **Demonstration of installation and usage of the SBM GridBean: Part 3.** This video shows how to submit and monitor the simulation and view the results. (MP4 13 MB)

## Abbreviations

API: Application programming interface
CML: Chemical markup language
GUI: Graphical user interface
HPC: High performance computing
HTC: High throughput computing
IDB: Incarnation data base
IT: Information technology
JSDL: Job submission description language
MD: Molecular dynamics
MDP: Molecular dynamics parameter
MSML: Molecular simulation markup language
NMR: Nuclear magnetic resonance
PDB: Protein data bank
RNA: Ribonucleic acid
SBM: Structure based modeling
SMOG: Structure-based MOdels in GROMACS
SOA: Service oriented architecture
SSL: Secure socket layer
URC: UNICORE Rich Client
XML: Extensible markup language.
